# Smoking history influences the prognostic value of peripheral naïve CD4+ T cells in advanced non-small cell lung cancer

**DOI:** 10.1186/s12935-019-0899-6

**Published:** 2019-07-09

**Authors:** Chao Liu, Bin Xu, Qian Li, Aijie Li, Lan Li, Jinbo Yue, Qinyong Hu, Jinming Yu

**Affiliations:** 1 Department of Oncology, Renmin Hospital of Wuhan University, Wuhan, 430060 China; 2 Department of Radiation Oncology, Shandong Cancer Hospital and Institute, Shandong First Medical University and Shandong Academy of Medical Sciences, Jinan, 250117 Shandong China; 3 Department of Radiation Oncology, Affiliated Hospital of Academy of Military Medical Sciences, Beijing, 100071 China; 4 Weifang Medical University, Weifang, 261053 Shandong China

**Keywords:** Memory T cells, Naïve T cells, Non-small cell lung cancer, Cigarette

## Abstract

**Background:**

Considering the effect of smoking on tumor immunity, we attempted to investigate the impact of smoking history on the prognostic value of circulating naïve and memory CD4+ and CD8+ T cells in advanced non-small cell lung cancer (NSCLC) treated with chemo(radio)therapy.

**Methods:**

Of 196 histologically confirmed advanced NSCLC, 98 eligible ones were enrolled. Naïve and memory CD4+ and CD8+ T cells from peripheral blood were measured by flow cytometry. Kaplan–Meier curves helped estimate patients’ survival. The uni- and multivariate Cox proportional hazards regression model was employed in the assessment of the prognostic value of factors.

**Results:**

Multivariate survival analyses showed that peripheral naïve CD4+ T cells independently predicted favorable overall survival (OS) in ever smokers with advanced NSCLC (P = 0.007), but unfavorable OS in never smokers with the same ailment (P = 0.012). Ever smokers presented a different distribution of naïve and memory T cells: low expression levels of naïve CD4+ T (P = 0.005), naïve CD8+ T (P = 0.031), CD4+ naïve/memory ratio (P = 0.020), and CD8+ naïve/memory ratio (P = 0.019), and high distributions of memory CD4 + T (P = 0.004), memory CD8 + T (P = 0.034), and naïve CD8/CD4 ratio (P = 0.020), when compared to never smokers.

**Conclusions:**

We revealed the impact of cigarette-smoking on peripheral naïve CD4+ T cells’ prognostic value in advanced NSCLC patients. These results could help in refining personalized treatment for advanced NSCLC patients.

**Electronic supplementary material:**

The online version of this article (10.1186/s12935-019-0899-6) contains supplementary material, which is available to authorized users.

## Background

Because of its aggressive tumor evasion and metastasis, lung cancer currently occupies the number one position in malignant tumor-related deaths worldwide [1–3]. Most patients with non-small cell lung cancer (NSCLC) get diagnosed with late stages, manifesting as local or systemic advanced diseases (stage III or IV), with an overall median survival of < 12 months [4].

Recently, inhibitors of immune checkpoints aimed at regulating programmed death-1 (PD-1)/PD-ligand 1 (PD-L1) have triggered extraordinary responses, becoming a new standard treatment for advanced NSCLC without targetable oncogenes [5–15]. Interestingly, ever smokers with NSCLC react to immune checkpoint inhibitors better than their nonsmoking counterparts, which may be explained by the following findings [16, 17]. Lung cancer in ever smokers features a high incidence of mutating somatic cells, including DNA repair gene mutations, an enormous load of neoantigens, and a stronger immunogenicity. On the contrary, never smokers are home to low mutational frequencies and an immunosuppressive tumor microenvironment [18–20]. Taking the tumor immune microenvironment differences between ever smokers and never smokers into consideration, several studies have investigated the impact of cigarette smoke on tumor infiltrating lymphocytes’ (TILs) prognostic role in NSCLC and established that the prognosis linking smoke to the subsets of TILs differed according to patients’ smoking history [21–24]. Inamura and his colleagues revealed that the high expression of B7-H3 in a tumor microenvironment was connected to reduced lung cancer-specific survival in moderate/heavy-smoking patients, but not in non/light-smoking patients [23]. Kinoshita et al. [24] also found a high ratio of forkhead box P3 (FOXP3)/CD4 to be of poor prognosis in relation to a smoking history, but not low levels of CD20+ B cells which were confirmed to be unfavorable to never smokers who had a complete resected NSCLC.

Because invasive procedures put patients at risks of complications, such as pneumothorax and bleeding [25–27], and because the scheduling of biopsies can impose significant treatment delays and logistical challenges for patients, obtain a tumor tissue to test for TILs, especially in patients with unresectable advanced NSCLC, has proven invasive. To make matters more complicated, clinicians frequently repeat biopsies for the purpose of optimizing their approaches to resistant disease. Consequently, interest is mounting in tumor profiling via the analysis of peripheral blood to avoid the dangers and inconveniences posed by potentially multiple invasive biopsy procedures.

The probable prognostic interaction between smoking and circulating lymphocyte subsets from peripheral blood in NSCLC, collected by the minimal invasive liquid biopsy, remains largely unknown. We hypothesized that smoking could influence the role of peripheral T cells in NSCLC and attempted to investigate the impact of smoking on the prognostic value of circulating naïve and memory lymphocyte subsets in advanced NSCLC treated with chemo(radio)therapy.

## Methods

### Study design

This study was carried out with the Ethics Committee of Affiliated Hospital of Academy of Military Medical Sciences’ approval. We obtained written informed consent from participating patients and healthy volunteers. Out of the 196 histological confirmed advanced NSCLC cases between February 2014 and December 2016, we enrolled 98 eligible patients treated with chemo(radio)therapy in the study. We excluded patients with incomplete clinicopathological data, known targetable oncogenes (anaplastic lymphoma kinase, epidermal growth factor receptor, and cMET), liver, hematological, and renal diseases, infection, performance status (PS) > 2, patients who received granulocyte-colony stimulating factor, steroids, and antilymphocyte globulin treatments within the 3 months that preceded enrollment, and patients with other tumors. Sixty-two age- and sex-matched healthy individuals were registered for control purposes.

### Clinical information

Data on age, smoking status, gender, histology, tumor stage matching the seventh American Joint Committee on Cancer (AJCC) staging system [28], tumor differentiation, and performance status were collected. Stage III patients received concurrent chemotherapy (cisplatin-based regime) and radiotherapy with 60–6 Gy/30–33 fractions. All stage IV patients underwent a cisplatin-based chemotherapy for 4–6 cycles. Follow-up was done regularly every 3 months and ended on October 2018.

### Sample collection and detection of naïve cells and memory T cells

Four milliliters of fresh peripheral blood were collected from patients 3 days before all treatment and healthy volunteers, and stored in EDTA anti-coagulant tubes. Peripheral blood cells were mixed with specific anti-human monoclonal antibodies (BD Biosciences; USA) against CD3 PerCP (cat. no. 552851), CD4 APC (cat. no. 555349), CD8 APC (cat. no. 555369), CD45RA FITC (cat. no. 555488), CD197 PE (CCR7, cat. no. 560765), and CD45RO PE (cat. no. 555493), along with the isotype antibody that served as the negative control, for 15 min in the dark at room temperature. Then we used Red Blood Cell lysing buffer (BD Biosciences; USA) to lyse red cells for 10 min in the dark at room temperature and flow cytometry (BD Biosciences; USA) to analyze residual white blood cells. The following naïve and memory T cells: naïve CD4+ T (CD3+CD4+CD45RA+CCR7+), memory CD4+ T (CD3+CD4+CD45RA−CD45RO+), naïve CD8+ T (CD3+CD8+CD45RA+CCR7+), and memory CD8+ T (CD3+CD8+CD45RA−CD45RO+) cells were identified. The data analysis software, FlowJo Version 10 (FlowJo, Ashland, OR, USA) was used to calculate the amounts of naïve and memory T cells. Representative flow cytometry plots and gating are presented in Additional file 1: Figure S1.

### Statistical analysis

Cut-off values for high or low naïve and memory T cells were determined by their respective median counts. For sub-group analyses of ever smokers and never smokers, we determined their cut-off values using the median counts of naïve and memory T cells in each sub-group. Progression-free survival marked the time from enrollment to the recurrence of a tumor, end or loss of follow-up, and death. Overall survival (OS) represented the interval between selection for participation and death and end or loss of follow-up. Analysis of data were undertaken using the SPSS 23.0 software (SPSS Inc., Chicago, IL). T cells were reported as mean ± standard deviation. The assessment of the correlation between T cell levels and clinical information and the comparison of T cells counts between patients and healthy controls were performed using the student’s t test. The univariate and multivariate Cox proportional hazards regression model helped with the evaluation of the hazard ratio (HR) and 95% confidence interval (CI). Variables with univariate analytical outcomes with *P *< 0.010 were passed on for multivariate analysis. We estimated patients’ survival with the use of the Kaplan–Meier curve and compared survival between groups using the Log-rank test. Statistical significance was considered at *P* value < 0.05.

## Results

### Patient characteristics

The baseline features of 98 advanced NSCLC cases are outlined in Table 1. Patients’ median age was calculated at 61.5 (43–90) years. The male patient representation was 63 (64.3%) as against 35 (35.7%) for the female patients. Taking part in the study were 55 (56.1%) ever smokers and 43 (43.9%) never smokers. Patients with stage IV received cisplatin-based chemotherapy. Stage III patients received concurrent radiotherapy (60–66 Gy/30–33 fractions) and cisplatin-based chemotherapy. We estimated the follow-up mean and median times at 15.5 and 13.3 months. 58 (59.2%) patients had died by the end of the last follow-up.

**Table 1:** Baseline characteristics of 98 advanced NSCLC patients

Characteristic	N (%)
Age (years)	
≥ 60	56 (57.1%)
< 60	42 (42.9%)
Gender	
Male	63 (64.3%)
Female	35 (35.7%)
Performance status	
0	26 (26.5%)
1–2	72 (73.5%)
Smoking status	
Ever smoker	55 (56.1%)
Never smoker	43 (43.9%)
cStage	
IV	71 (72.4%)
III	27 (27.6%)
Histology	
AD	50 (51.0%)
Non-AD	48 (49.0%)
Tumor differentiation	
Poor	36 (36.7%)
Moderate	51 (52.0%)
Well	4 (4.1%)
None	7 (7.1%)

**Fig. 1:**
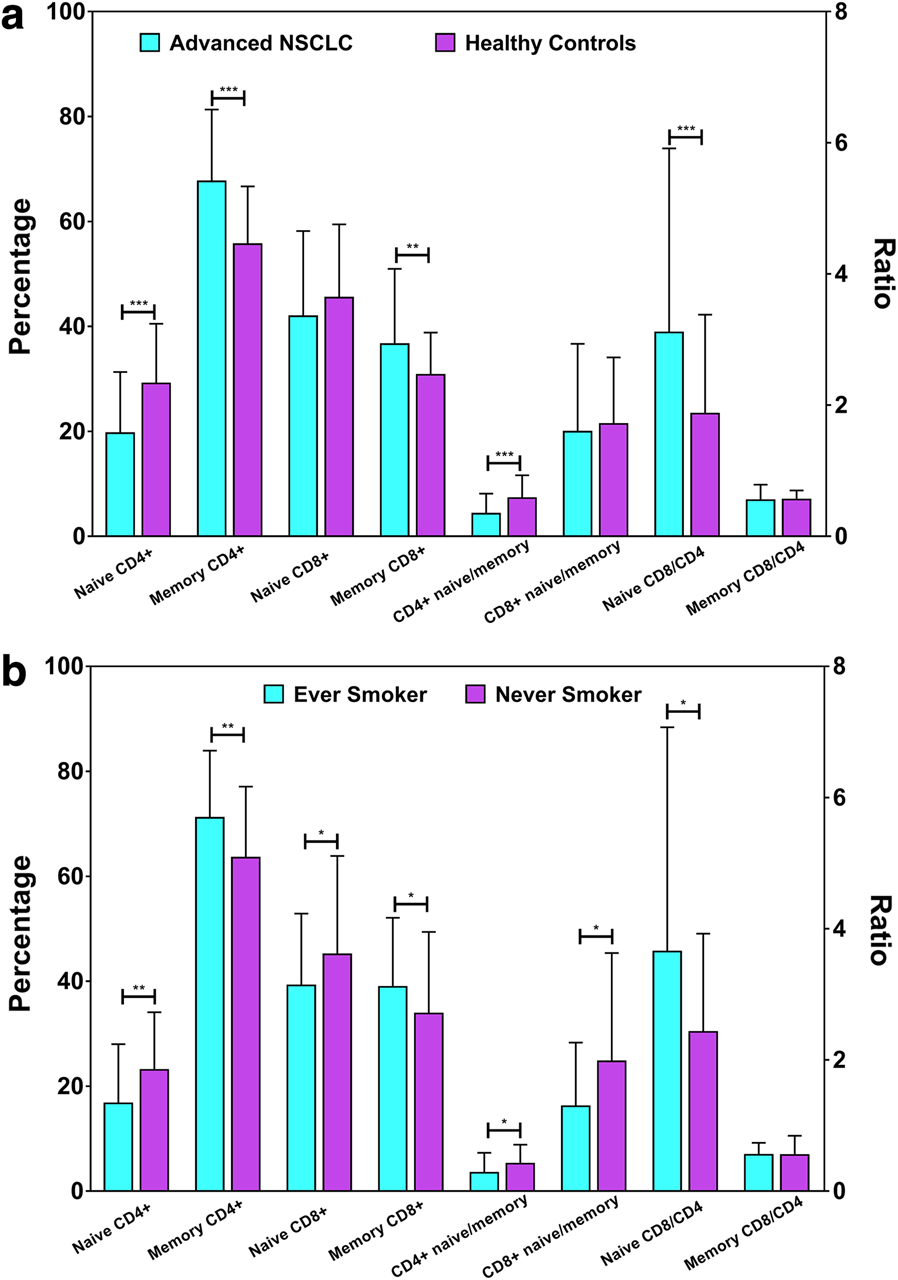
**a** Comparisons of naïve cells and memory T cells between 98 advanced NSCLC patients and 62 healthy controls. NSCLC patients had low levels of naïve CD4+ T (P < 0.001) and CD4+ naïve/memory ratio (P < 0.001), and high levels of memory CD4+ T (P < 0.001), memory CD8+ T (P = 0.001), and naïve CD8/CD4 ratio (P < 0.001) than healthy controls. **b** Comparisons of naïve cells and memory T cells between 55 ever smokers and 43 never smokers. Ever smokers expressed low levels of naïve CD4+ T (P = 0.005), naïve CD8+ T (P = 0.031), CD4+ naïve/memory ratio (P = 0.020) and CD8+ naïve/memory ratio (P = 0.019), and high memory CD4+ T (P = 0.004), memory CD8+ T (P = 0.034) counts, and naïve CD8/CD4 ratio (P = 0.020) than never smokers. Error bar represents standard deviation (SD). *P < 0.05, **P < 0.01, ***P < 0.001, two-sided paired Student’s t-test (**a**, **b**)

NSCLC patients had low levels of naïve CD4+ T (19.6 ± 11.6 vs. 29.1 ± 11.3, P < 0.001) and a low CD4+ naïve/memory ratio (0.3 ± 0.3 vs. 0.6 ± 0.3, P < 0.001) and high levels of memory CD4+ T (67.6 ± 13.6 vs. 55.6 ± 11.0, P < 0.001) and memory CD8 + T (36.6 ± 14.3 vs. 30.7 ± 8.0, P = 0.001), as well as a high naïve CD8/CD4 ratio (3.1 ± 2.8 vs. 1.8 ± 1.5, P < 0.001) than healthy controls (Fig. 1a).

### Difference in the distribution of naïve and memory T cells between ever smokers and never smokers

The expression of immune cells greatly differed between ever smokers and never smokers (Fig. 1b) but not in the comparisons between other parameters (Additional file 1: Figure S2). Ever smokers expressed low levels of naïve CD4+ T (16.7 ± 11.3 vs. 23.4 ± 11.1, P = 0.005) and naïve CD8+ T (39.2 ± 13.7 vs. 45.4 ± 18.6, P = 0.031), as well as a low CD4+ naïve/memory ratio (0.3 ± 0.3 vs. 0.4 ± 0.3, P = 0.020), and a low CD8+ naïve/memory ratio (1.3 ± 1.0 vs. 2.0 ± 1.6, P = 0.019) than never smokers. Ever smokers also had high memory CD4+ T (71.1 ± 12.8 vs. 63.1 ± 13.6, P = 0.004) and memory CD8+ T (38.9 ± 13.1 vs. 33.6 ± 15.4, P = 0.034) counts, as well as a high naïve CD8/CD4 ratio (3.7 ± 3.4 vs. 2.4 ± 1.5, P = 0.020) than never smokers (Fig. 1b). These findings suggest that naïve and memory T cells were quite differently distributed between ever smokers and never smokers.

### Naïve CD4+ T cells’ favorable prognostic value in ever smokers

To determine, for all of 98 advanced NSCLC cases, if the OS and DFS of patients with high immune cells were significantly different from those of patients with low immune cells, the Kaplan–Meier analysis was performed, with results shown in Additional file 1: Figure S3–S4. Consequently, we found that there was no significant correlation between immune cells and survival (all P > 0.05).

**Fig. 2:**
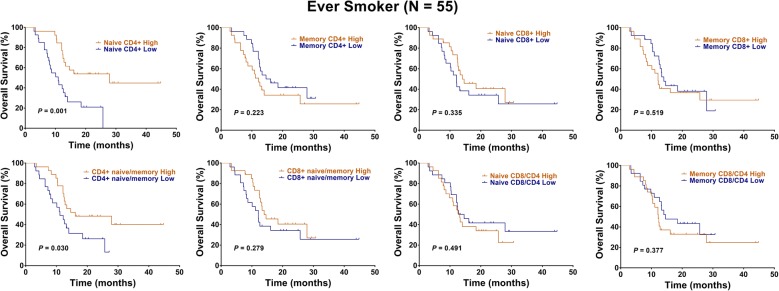
Kaplan–Meier representations of OS with respect to the distribution of naïve CD4+ T, memory CD4+ T, naïve CD8+ T, memory CD8+ T, CD4+ naïve/memory ratio, CD8+ naïve/memory ratio, naïve CD8/CD4 ratio, and memory CD8/CD4 ratio in 55 ever smokers. High levels of naïve CD4+ T cells (P = 0.001) and a high CD4+ naïve/memory ratio (P = 0.030) predicted a better OS. Log-rank test

**Fig. 3:**
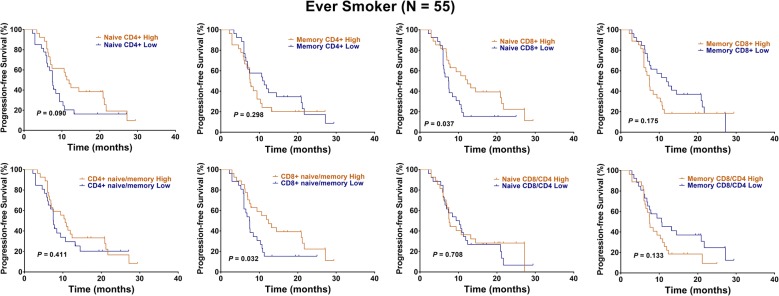
Kaplan–Meier representations of PFS with respect to the distribution of naïve CD4+ T, memory CD4+ T, naïve CD8+ T, memory CD8+ T, CD4+ naïve/memory ratio, CD8+ naïve/memory ratio, naïve CD8/CD4 ratio, and memory CD8/CD4 ratio in 55 ever smokers. High levels of naïve CD8+ T cells (P = 0.037) and a high CD8+ naïve/memory ratio (P = 0.032) predicted a better PFS. Log-rank test

However, considering of the distinct naïve and memory T cell levels between ever smokers and never smokers, we performed subgroup analyses. Interestingly, we found that high levels of naïve CD4+ T cells (HR: 0.35, 95% CI 0.17–0.70, P = 0.001, Fig. 2, Table 2) and a high CD4+ naïve/memory ratio (HR: 0.48, 95% CI 0.24–0.97, P = 0.030, Fig. 2, Table 2) predicted a better OS in 55 ever smokers. In addition, high levels of naïve CD8+ T cells (HR: 0.54, 95% CI 0.29–1.01, P = 0.037, Fig. 3, Table 2) and a high CD8+ naïve/memory ratio (HR: 0.53, 95% CI 0.28–0.99, P = 0.032, Fig. 3, Table 2) predicted a better PFS. Multivariate analysis showed that the presence of naïve CD4+ T cells independently predicted a favorable OS in ever smokers (HR: 0.11, 95% CI 0.02–0.55, P = 0.007, Table 2).

**Table 2:** Cox regression analysis of survival for ever smokers among NSCLC patients

Variables	OS		PFS	
	HR (95% CI)	P value	HR (95% CI)	P value
Univariate analysis				
Age (years)				
< 60	1		1	
≥ 60	1.29 (0.64–2.58)	0.468	1.25 (0.66–2.34)	0.488
Gender				
Female	1		1	
Male	1.26 (0.30–5.30)	0.746	1.22 (0.36–4.08)	0.747
Performance status				
0	1		1	
1–2	1.76 (0.76–4.04)	0.18	1.41 (0.70–2.80)	0.331
cStage				
III	1		1	
IV	1.35 (0.66–2.75)	0.404	1.84 (0.96–3.53)	0.066
Histology				
Non-AD	1		1	
AD	1.37 (0.70–2.66)	0.352	1.71 (0.93–3.15)	0.183
Tumor differentiation				
Well/moderate	1		1	
Poor	1.58 (0.80–3.12)	0.187	1.14 (0.61–2.14)	0.677
Naive CD4+				
Low	1		1	
High	0.35 (0.17–0.70)	0.001	0.60 (0.32–1.12)	0.09
Memory CD4+				
Low	1		1	
High	1.51 (0.76–2.98)	0.223	1.36 (0.73–2.51)	0.298
Naive CD8+				
Low	1		1	
High	0.72 (0.36–1.41)	0.335	0.54 (0.29–1.01)	0.037
Memory CD8+				
Low	1		1	
High	1.24 (0.63–2.44)	0.519	1.50 (0.81–2.77)	0.175
CD4+ naive/memory				
Low	1		1	
High	0.48 (0.24–0.97)	0.03	0.78 (0.42–1.44)	0.411
CD8+ naive/memory				
Low	1		1	
High	0.69 (0.35–1.36)	0.279	0.53 (0.28–0.99)	0.032
Naive CD8/CD4				
Low	1		1	
High	1.26 (0.64–2.47)	0.491	0.89 (0.48–1.63)	0.708
Memory CD8/CD4				
Low	1		1	
High	1.35 (0.68–2.64)	0.377	1.56 (0.84–2.87)	0.133
Multivariate analysis				
Naive CD4+				
Low	1		1	
High	0.11 (0.02–0.55)	0.007	0.73 (0.35–1.52)	0.400
CD4+ naive/memory				
Low	1		–	
High	3.63 (0.77–16.95)	0.101	–	–
Naive CD8+				
Low	–		1	
High	–	–	0.69 (0.11–4.17)	0.693
CD8+ naive/memory				
Low	–		1	
High	–	–	0.80 (0.12–5.03)	0.815
cStage				
III	–		1	
IV	–	–	1.97 (0.99–3.92)	0.051

### Naïve CD4+ T cells’ unfavorable prognostic value in never smokers

**Fig. 4:**
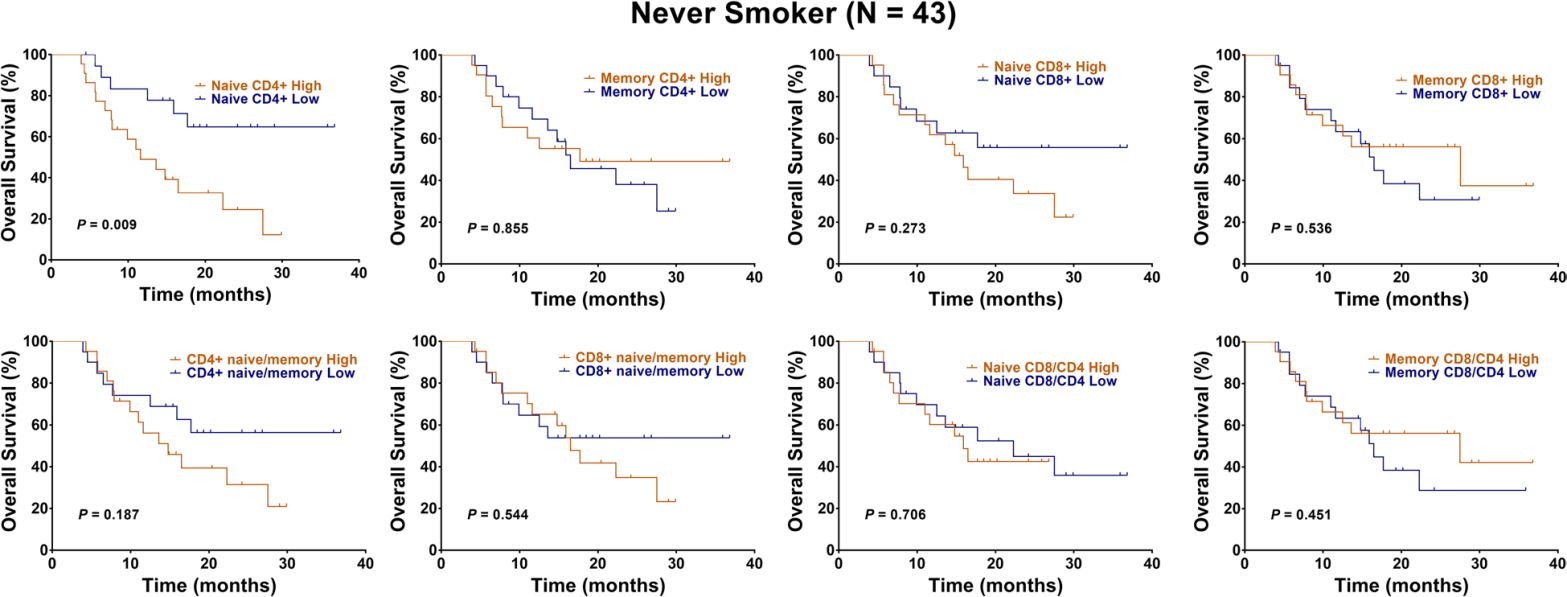
Kaplan–Meier representations of OS with respect to the distribution of naïve CD4+ T, memory CD4+ T, naïve CD8+ T, memory CD8+ T, CD4+ naïve/memory ratio, CD8+ naïve/memory ratio, naïve CD8/CD4 ratio, and memory CD8/CD4 ratio in 43 never smokers. High levels of naïve CD4+ T cells predicted a shorter OS (P = 0.009). Log-rank test

**Fig. 5:**
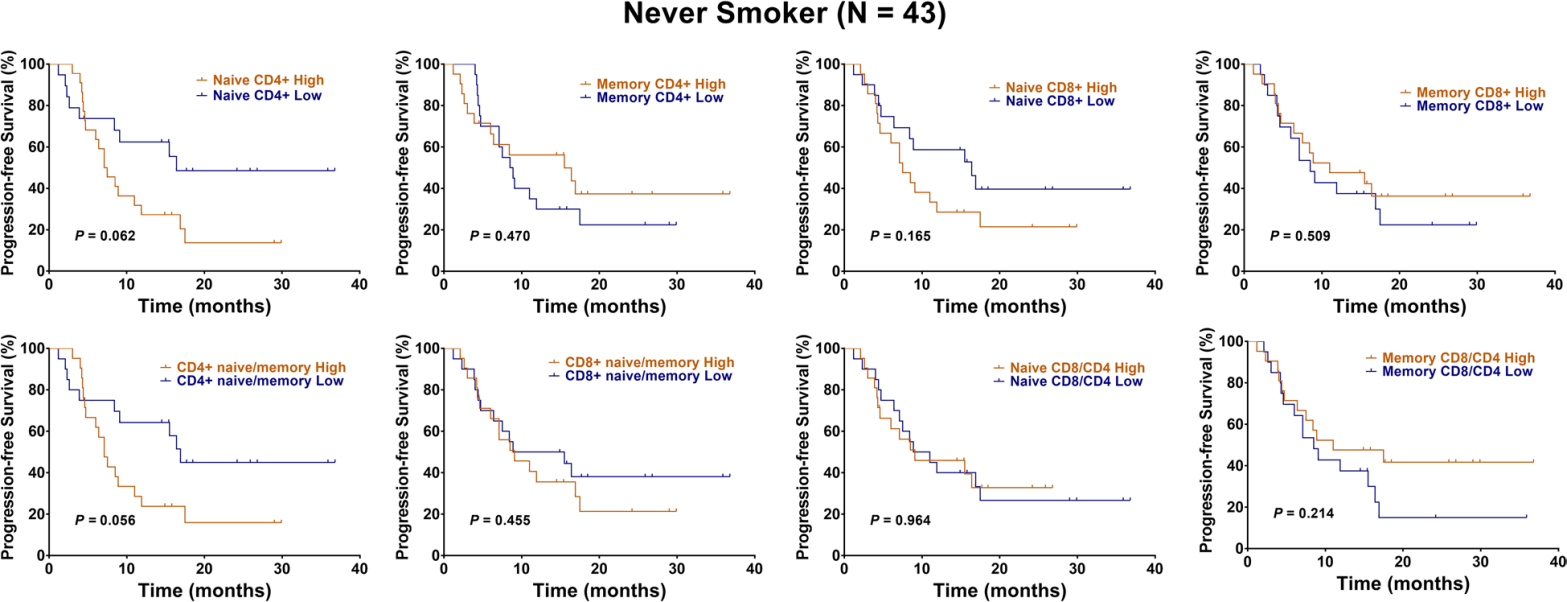
Kaplan–Meier representations of PFS with respect to the distribution of naïve CD4+ T, memory CD4+ T, naïve CD8+ T, memory CD8+ T, CD4+ naïve/memory ratio, CD8+ naïve/memory ratio, naïve CD8/CD4 ratio, and memory CD8/CD4 ratio in 43 never smokers. High levels of naïve CD4+ T cells and CD4+ naïve/memory ratio were associated with a poor PFS, with strong trend (P = 0.062 and 0.056). Log-rank test

Kaplan–Meier analysis and univariate analysis showed that high levels of naïve CD4+ T cells predicted a shorter OS in each of 43 never smokers (HR: 3.16, 95% CI 1.38–7.40, P = 0.009, Fig. 4, Table 3). Besides, high levels of naïve CD4+ T cells and CD4+ naïve/memory ratio were associated with a poor PFS, with strong trend (HR: 2.07, 95% CI 0.97–4.41, P = 0.062; HR: 2.07, 95% CI 0.97–4.42, P = 0.056, Fig. 5, Table 3). Multivariate analysis showed that naïve CD4+ T cells independently predicted unfavorable OS in never smokers (HR: 2.17, 95% CI 1.15–5.49, P = 0.012, Table 3).

**Table 3:** Cox regression analysis of survival for never smokers among NSCLC patients

Variables	OS		PFS	
	HR (95% CI)	P value	HR (95% CI)	P value
Univariate analysis				
Age (years)				
< 60	1		1	
≥ 60	0.46 (0.19–1.09)	0.081	0.44 (0.21–0.95)	0.036
Gender				
Female	1		1	
Male	1.81 (0.76–4.32)	0.18	2.06 (0.94–4.52)	0.07
Performance status				
0	1		1	
1–2	1.36 (0.39–4.70)	0.625	1.32 (0.50–3.50)	0.568
cStage				
III	1		1	
IV	0.98 (0.36–1.70)	0.981	0.73 (0.32–1.65)	0.451
Histology				
Non-AD	1		1	
AD	0.87 (0.35–3.15)	0.77	0.81 (0.36–1.79)	0.61
Tumor differentiation				
Well/moderate	1		1	
Poor	1.86 (0.76–4.50)	0.169	2.74 (1.22–6.14)	0.014
Naive CD4+				
Low	1		1	
High	3.19 (1.38–7.40)	0.009	2.07 (0.97–4.41)	0.062
Memory CD4+				
Low	1		1	
High	0.92 (0.40–2.13)	0.855	0.75 (0.35–1.61)	0.47
Naive CD8+				
Low	1		1	
High	1.61 (0.69–3.72)	0.273	1.70 (0.79–3.62)	0.165
Memory CD8+				
Low	1		1	
High	0.76 (0.33–1.77)	0.536	0.77 (0.36–1.65)	0.509
CD4+ naive/memory				
Low	1		1	
High	1.77 (0.76–4.08)	0.187	2.07 (0.97–4.42)	0.056
CD8+ naive/memory				
Low	1		1	
High	1.29 (0.56–2.99)	0.544	1.33 (0.62–2.83)	0.455
Naive CD8/CD4				
Low	1		1	
High	1.16 (0.50–2.70)	0.706	0.98 (0.46–2.09)	0.964
Memory CD8/CD4				
Low	1		1	
High	0.72 (0.31–1.68)	0.451	0.62 (0.29–1.33)	0.214
Multivariate analysis				
Naive CD4+				
Low	1		1	
High	2.17 (1.15–5.49)	0.012	2.43 (0.22–26.80)	0.468
CD4+ naive/memory				
Low	–			
High	–	–	0.82 (0.09–7.18)	0.858
Age (years)				
< 60	1		1	
≥ 60	0.47 (0.19–1.13)	0.094	0.70 (0.28–1.74)	0.447
Gender				
Female	–		1	
Male	–	–	1.79 (0.72–4.48)	0.209
Tumor differentiation				
Well/moderate	–		1	
Poor	–	–	2.07 (0.84–5.08)	0.112

## Discussion

The results of this study confirmed our hypothesis that smoking influences naïve and memory T cells’ prognostic value in patients with advanced NSCLC. Intriguingly, we found that the presence of peripheral naïve CD4+ T cells had a favorable prognostic impact for ever smokers, but an unfavorable prognostic value for never smokers. We also revealed a distinctive distribution of naïve and memory T cells between ever smokers and never smokers. Ever smokers expressed low levels of naïve CD4+ T and naïve CD8+ T, as well as a low CD4+ naïve/memory ratio and a low CD8+ naïve/memory ratio and high levels of memory CD4+ T and memory CD8+ T, as well as a high naïve CD8/CD4 ratio than never smokers. These results could provide evidence for an interesting interactive significance between naïve and memory T cells and smoking in advanced NSCLC.

Two previous investigations have examined the prognostic value of naïve and memory T cells in resectable NSCLC [29, 30]. Yang and his colleagues reported that an increased CD4+ naive/memory ratio predicted better PFS in 76 resectable NSCLC [30]. Hara et al. [29] figured out that the CD4(+) naive/memory ratio had a prognostic relevance in 38 patients with NSCLC that underwent surgery. They proved the possible association between peripheral naïve cells and peripheral memory T cells and survival in NSCLC. However, they did not examine the interaction of naïve cells and memory cells with smoking history. Additionally, the sample size used by these two studies was too small, which may explain why they did not find any differences in naïve and memory cells between ever smokers and never smokers.

In our study, we enrolled 98 patients with advanced NSCLC and found a significant difference in naïve and memory T cells’ distribution between ever smokers and never smokers, which has not been reported in any studies before. Smoking induces a high incidence of mutations in somatic cells, including DNA repair genes’ mutations, a huge load of neoantigens [18, 19, 31] that activated naïve T cells’ differentiation into effector T cells to eliminate the neoantigens [32–36]. These findings support our results that lower levels of naïve CD4+ T and naïve CD8+ T, as well as a lower CD4+ naïve/memory ratio and a lower CD8+ naïve/memory ratio, together with higher levels of memory CD4+ T and memory CD8+ T existed in ever smokers compared to never smokers.

We report the contrasting naïve CD4+ T cells’ prognostic values between ever smokers and never smokers with advanced NSCLC. To date, no investigative studies have described this phenomenon. Tobacco smoking creates a high frequency of somatic mutations, a huge burden of neoantigens, and an amplified immunogenicity, which possibly maintain the greater proliferative potential of naïve CD4+ cells and CD8+ T cells that have been linked to greater immune efficacy [37]. These findings back our results that naïve CD4+ T cells’ prognostic value was favorable in ever smokers with NSCLC. On the other hand, never smokers harbor a low burden of mutation and immunosuppressive feature, quite possibly a contributing factor in naive CD4+ T cells’ differentiation into immunosuppressive CD4+ Treg [34], which back our findings of naïve CD4+ T cells’ unfavorable prognostic value in never smokers with NSCLC.

Some limitations exist in our study. First, the 98 advanced NSCLC constitutes a small sample pool. Second, subgroup analyses of prognostic values in ever smokers and never smokers based on histological types were not performed because of the limited sample size. Further studies are needed to address this issue. Third, exposure to smoking by never smokers was not considered since the smoking status used depended fully on patients’ self-evaluation. Finally, we did not explore the underlying mechanisms for our findings. Further studies are needed to investigate the underlying mechanisms. Despite these limitations, our results suggest opposing values of prognosis of naïve CD4+ T cells between ever smokers and never smokers with advanced NSCLC.

## Conclusions

We revealed the impact of cigarette smoking on the prognostic values of naïve and memory T cells in advanced NSCLC patients. Peripheral naïve CD4+ T cells had a favorable prognostic significance in ever smokers, but an unfavorable prognostic value in never smokers. We also found a significant difference in naïve and memory T cells’ distribution between ever smokers and never smokers. These results could help refine personalized treatment for advanced NSCLC.

## Additional file

**Additional file 1: Figure S1.** Representative plots for flow cytometry analysis showing (A) naïve CD4+ T and memory CD4+ T; (B) naïve CD8+ T and memory CD8+ T cells. FlowJo Version 10 (FlowJo, Ashland, OR, USA) was used to evaluate naïve and memory T cells. **Figure S2.** Comparisons of naïve cells and memory T cells between AD and Non-AD, female and male, age < 60 and age ≥ 60, ECOG PS 0 and 1–2, stages III and IV, and poor and moderate/good differentiation. Error bar represents SD. *P < 0.05, **P < 0.01, ***P < 0.001, two-sided paired Student’s t-test. **Figure S3.** Kaplan–Meier representations of OS with the respect to the distribution of naïve CD4+ T, memory CD4+ T, naïve CD8+ T, memory CD8+ T, CD4+ naïve/memory ratio, CD8+ naïve/memory ratio, naïve CD8/CD4 ratio, and memory CD8/CD4 ratio in 98 advanced NSCLC patients. No significant difference of OS between patients with high and low immune cells (all P > 0.05). Log-rank test. **Figure S4.** Kaplan–Meier representations of PFS with respect to the distribution of naïve CD4+ T, memory CD4+ T, naïve CD8+ T, memory CD8+ T, CD4+ naïve/memory ratio, CD8+ naïve/memory ratio, naïve CD8/CD4 ratio, and memory CD8/CD4 ratio in 98 advanced NSCLC patients. No significant difference of PFS between patients with high and low immune cells (all P > 0.05). Log-rank test.

## Data Availability

All data included in our study are shown in our manuscript.

## Abbreviations

NSCLC: non-small cell lung cancer
OS: overall survival
PD-1: programmed death-1
FOXP3: forkhead box P3
PFS: progression-free survival
