# Supplementary material for: Smoking history influences the prognostic value of peripheral naïve CD4+ T cells in advanced non-small cell lung cancer
Source: Cancer Cell Int. 2019 Jul 9;19:176. doi: 10.1186/s12935-019-0899-6 (PMC6617618; doi:10.1186/s12935-019-0899-6)
Supplement: Supplementary file 1 — Additional file 1: Figure S1. Representative plots for flow cytometry analysis showing (A) naïve CD4+ T and memory CD4+ T; (B) naïve CD8+ T and memory CD8+ T cells. FlowJo Version 10 (FlowJo, Ashland, OR, USA) was used to evaluate naïve and memory T cells. Figure S2. Comparisons of naïve cells and memory T cells between AD and Non-AD, female and male, age < 60 and age ≥ 60, ECOG PS 0 and 1–2, stages III and IV, and poor and moderate/good differentiation. Error bar represents SD. *P < 0.05, **P < 0.01, ***P < 0.001, two-sided paired Student’s t-test. Figure S3. Kaplan–Meier representations of OS with the respect to the distribution of naïve CD4+ T, memory CD4+ T, naïve CD8+ T, memory CD8+ T, CD4+ naïve/memory ratio, CD8+ naïve/memory ratio, naïve CD8/CD4 ratio, and memory CD8/CD4 ratio in 98 advanced NSCLC patients. No significant difference of OS between patients with high and low immune cells (all P > 0.05). Log-rank test. Figure S4. Kaplan–Meier representations of PFS with respect to the distribution of naïve CD4+ T, memory CD4+ T, naïve CD8+ T, memory CD8+ T, CD4+ naïve/memory ratio, CD8+ naïve/memory ratio, naïve CD8/CD4 ratio, and memory CD8/CD4 ratio in 98 advanced NSCLC patients. No significant difference of PFS between patients with high and low immune cells (all P > 0.05). Log-rank test. [file 12935_2019_899_MOESM1_ESM.docx]

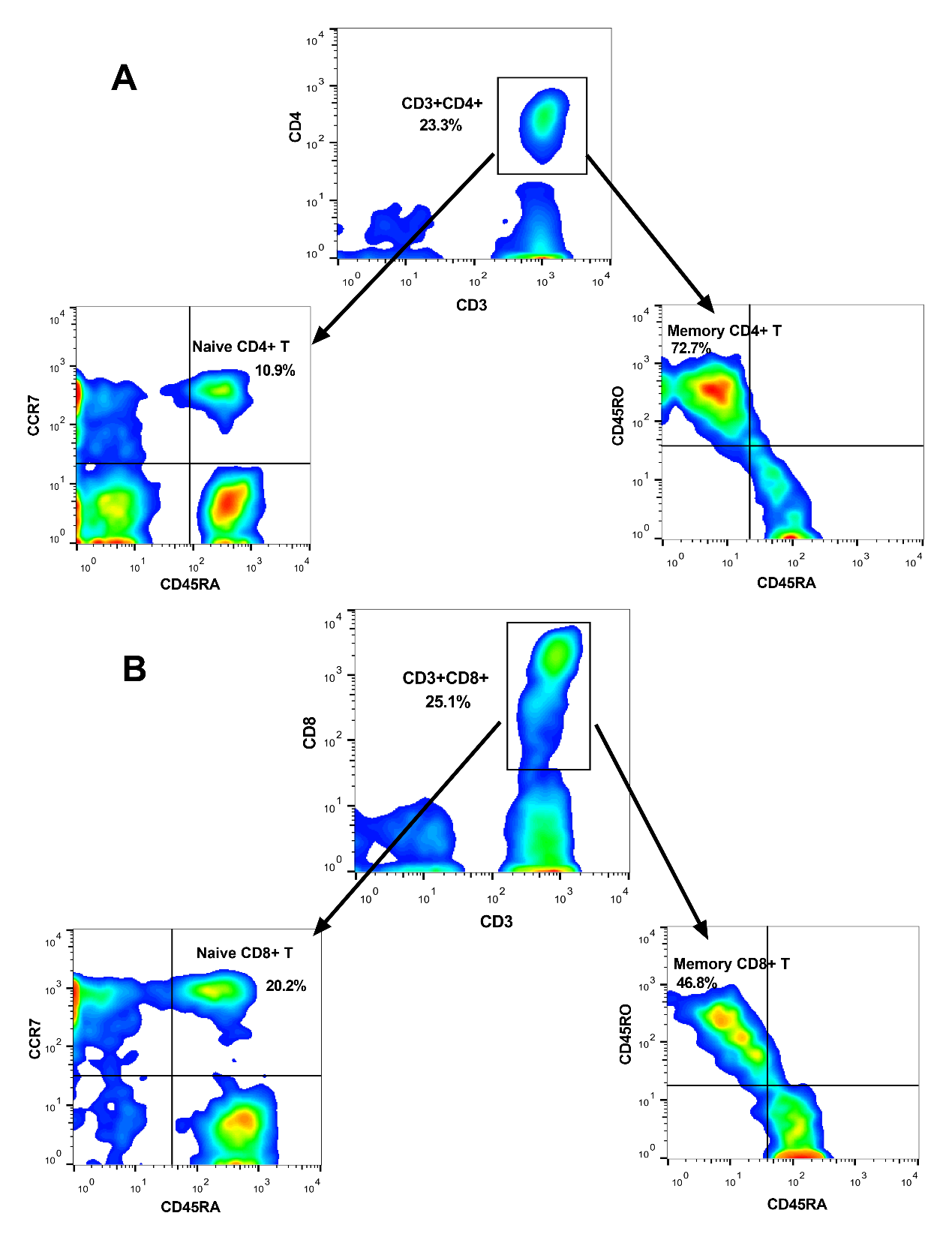


**Figure S1.** Representative plots for ﬂow cytometry analysis showing (A) naïve CD4+ T and memory CD4+ T; (B) naïve CD8+ T and memory CD8+ T cells. FlowJo Version 10 (FlowJo, Ashland, OR, USA) was used to evaluate naïve and memory T cells.


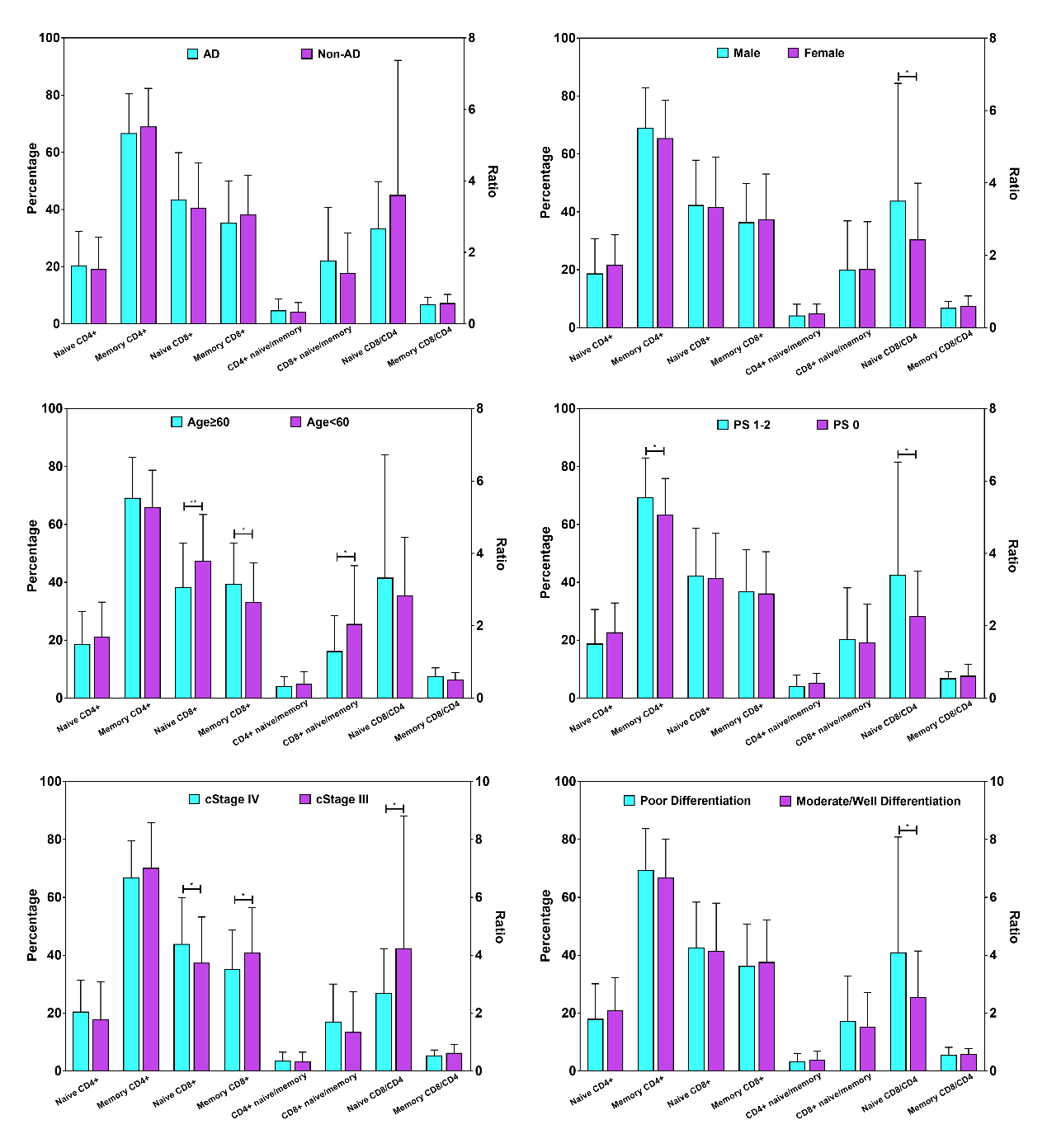


**Figure S2.** Comparisons of naïve cells and memory T cells between AD and Non-AD, female and male, age < 60 and age ≥ 60, ECOG PS 0 and 1-2, stages III and IV, and poor and moderate/good differentiation. Error bar represents SD. *P < 0.05, **P < 0.01, ***P < 0.001, two-sided paired Student’s t-test.


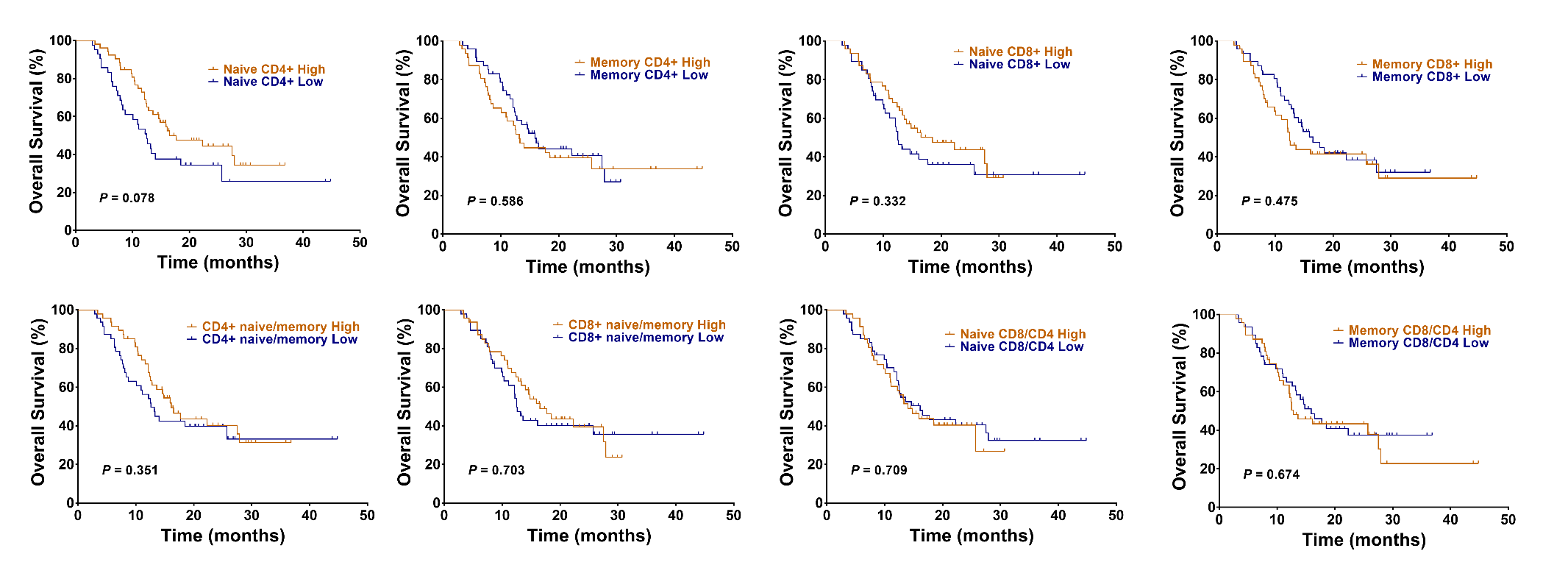


**Figure S3.** Kaplan-Meier representations of OS with the respect to the distribution of naïve CD4+ T, memory CD4+ T, naïve CD8+ T, memory CD8+ T, CD4+ naïve/memory ratio, CD8+ naïve/memory ratio, naïve CD8/CD4 ratio, and memory CD8/CD4 ratio in 98 advanced NSCLC patients. No significant difference of OS between patients with high and low immune cells (all P > 0.05). Log-rank test.


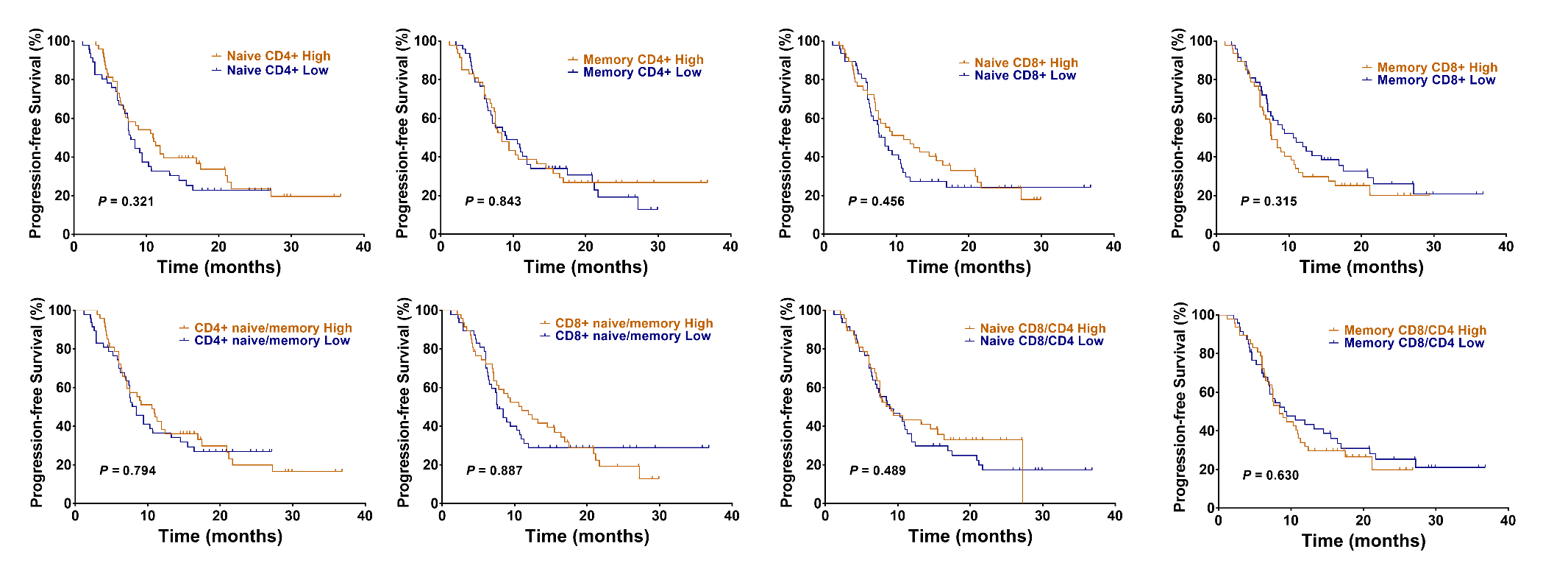


**Figure S4.** Kaplan-Meier representations of PFS with respect to the distribution of naïve CD4+ T, memory CD4+ T, naïve CD8+ T, memory CD8+ T, CD4+ naïve/memory ratio, CD8+ naïve/memory ratio, naïve CD8/CD4 ratio, and memory CD8/CD4 ratio in 98 advanced NSCLC patients. No significant difference of PFS between patients with high and low immune cells (all P > 0.05). Log-rank test.
